# The effect of COVID-19 vaccination on serum levels of anti-Müllerian hormone in women of reproductive age

**DOI:** 10.5935/1518-0557.20240098

**Published:** 2025

**Authors:** Fernanda Godoy Cabral de Oliveira, Renato de Oliveira, Bianca Bianco, Michel Soane, Cláudia Fideles, Nathalia Saraiva, Denise Christofolini, Caio Parente Barbosa

**Affiliations:** 1 Pós-Graduação em Ciências da Saúde do Centro Universitário FMABC, Santo André, Brazil; 2 Disciplina de Saúde Sexual, Reprodutiva e Genética Populacional, Departamento de Saúde da Coletividade, Centro Universitário FMABC, Santo André, Brazil; 3 Departamento de Pesquisa & Desenvolvimento (EUROInstitute), EUROIMMUN Brasil Medicina Diagnóstica, São Caetano do Sul, Brazil

**Keywords:** serological test for COVID-19, anti-Müllerian hormone, ovarian reserve, reproductive health, COVID-19 vaccines

## Abstract

**Objective:**

To evaluate the effects of COVID-19 vaccination (AstraZeneca^®^ and CoronaVac^®^) on anti-Müllerian hormone (AMH) levels in threatened women.

**Methods:**

Retrospective cohort study evaluating serum AMH before and up to three vaccination doses against COVID-19 between 2021 and 2022 at FMABC. Statistical analysis presented in Stata 14. Clinical variables were described by absolute and relative frequency, in addition to measures of central tendency and dispersion. Shapiro-Wilk test for normality. Continuous variables compared within the group using the Friedman test and, between groups, Mann-Whitney U tests (non-parametric); Chi-square and Fisher’s exact tests, for categorical variables, with *p*<0.05.

**Results:**

Median age of the 38 volunteers was 24 years (p25-75: 22-30) and AMH levels (ng/dl) at times 0, 1, 2 and 3 median (95% CI) were, respectively, 4.6(3.5-6); 4(2.3-5); 4.3(3-5); 4.9(2.6-6.3), *p*=0.726. Likewise, there was no statistically significant difference in the assessments between subgroups aged <35 and ≥35 years old and with and without exposure to COVID-19 in relation to AMH values.

**Conclusions:**

The vaccination against COVID-19 with the AstraZeneca^®^ and CoronaVac^®^ vaccines did not indicate any damage to anti-Müllerian hormone values in women of reproductive age.

## INTRODUCTION

Since the start of the COVID-19 pandemic in Wuhan, caused by the severe acute respiratory syndrome coronavirus 2 (SARS-CoV-2), nearly 200 million people have been infected, leading to more than seven million deaths (World Health Organization, 2024). As the pandemic progressed, several manifestations unrelated to respiratory symptoms were observed, including those of the reproductive system (Ding *et al*., 2021).

However, it is still uncertain what consequences COVID-19 would have on both female reproductive function and the hypothalamic-pituitary-gonadal axis. Thus, assessing ovarian reserve is one way to advance this issue. Defined as the set of ovarian follicles available for follicular recruitment, reflecting the functional potential of the ovaries (Chuang *et al*., 2003; Maheshwari *et al*., 2006), it can be considered a complex clinical phenomenon influenced by age, genetic and environmental variables (Tal & Seifer, 2013).

Among the biomarkers of ovarian reserve, the dosage of anti-Müllerian hormone (AMH) stands out, considered the most sensitive, allowing its assessment at any time during the menstrual cycles (Tal & Seifer, 2017).

Women affected by SARS-CoV-2 had lower AMH levels, suggesting a decrease in ovarian reserve. This has raised concerns about the potential deleterious impact of SARS-CoV-2 infection on fertility (Ding *et al*., 2021).

However, during the pandemic, vaccination has become an important ally in reducing mortality from COVID-19, contributing to the population’s hope of overcoming this health and humanitarian crisis. Regarding AMH monitoring and vaccination against COVID-19, studies in several locations such as the Czech Republic, Israel, and Turkey, using vaccines such as Pfizer/BioNTech COVID-19 Vaccine^®^ and Moderna® have shown that AMH levels with up to two doses did not have a statistically significant difference (Kolatorova *et al*., 2022; Mohr-Sasson *et al*., 2022; Horowitz *et al*., 2022; Soysal & Yilmaz, 2022).

In Brazil, the first reported case of COVID-19 occurred on February 26, 2020, in the city of São Paulo, after the arrival of a Brazilian from Italy (Gadelha *et al*., 2020). In the same year, 58 vaccines against SARS-CoV-2 were developed. Among them, the Oxford-AstraZeneca^®^ vaccine stands out, a more affordable vaccine aimed at global equity and commitment to lowand middle-income countries (Knoll & Wonodi, 2021). However, since September 2021, the most widely used vaccine for immunizing the elderly and health professionals in Brazil has been the inactivated SARS-CoV-2 vaccine CoronaVac^®^ (Sinovac Life Sciences; Beijing, China; Volpe *et al*., 2023).

To our knowledge, no study has evaluated ovarian reserve, represented by AMH values, in Brazilian patients who received the AstraZeneca^®^ and CoronaVac^®^ vaccines, which are widely used throughout the country. Considering the global repercussions of this COVID-19 pandemic, assessing this potential reproductive safety is of public health interest.

Thus, the objective of the research consists of the null hypothesis that women of reproductive age who received the AstraZeneca^®^ and CoronaVac^®^ vaccines for COVID-19 did not present changes in the ovarian reserve marker AMH, and the alternative hypothesis that vaccination led to a decrease in AMH.

## MATERIAL AND METHODS

### Study Design, Setting, and Participants

This retrospective cohort study was conducted at the ABC Medical School University Center (Centro Universitário FMABC) with workers who had no verbal reports of immunodeficiency, coming from regional hospitals and institutions, and who were about to receive the first dose of AstraZeneca^®^ or CoronaVac^®^ vaccines between August 1, 2021, and January 31, 2022. The STROBE recommendations (von Elm *et al*., 2008) were followed in this research.

Inclusion criteria were professionals from the Centro Universitário FMABC, aged between 18 and 40 years, not currently pregnant, in their reproductive years, and who received at least two doses of the COVID-19 vaccine after the collection without a vaccine.

Exclusion criteria included a history of ovarian endometriosis, previous ovarian surgery, report of infertility, a family history of premature ovarian insufficiency, reports of polycystic ovary syndrome, previous use of COVID-19 vaccine, and a history of radiotherapy or chemotherapy.

The serum samples used in this study were the same previously used in the study titled “Evaluation of humoral and T-cell-based responses after SARS-CoV-2 vaccination”, conducted between 2021 and 2022, which was approved by the ethics committee of this institution (Approval: 4.702.022). However, only samples from patients who met the aforementioned criteria were included.

### Variables (Data Collection and Samples)

Data collected included age, prior exposure to SARS-CoV-2, vaccination date, and the name and manufacturer of the vaccine received by the volunteer. According to prior exposure confirmed by serology or real-time reverse transcription-polymerase chain reaction (RT-PCR) to SARS-CoV-2, two groups were formed: exposed (EXP) and non-exposed (NEXP) for statistical analyses. Similarly, age < 35 years and ≥ 35 years were considered, given the impact on ovarian reserve with advancing age (Ghaemi *et al*., 2024) for subgroup analyses.

Biological material was collected at three time points: T0: immediately before the vaccine administration; T1: about 14 days after the first dose of the vaccine; T2: about 14 days after the second dose of the vaccine; T3: approximately 14 days after the booster dose. This totaled a period of 9 months between the initial collection from the first volunteer and the final collection from the last volunteer (Table 1).

**Table 1:** Timeline of serum collections for AMH^*^ analysis.

Peripheral blood sample	Pré-vaccine	2 weeks after 1st vaccine dose	2 weeks after 2nd vaccine dose	2 weeks after 3rd vaccine dose
Times	T0	T1	T2	T3

* AMH: Anti-müllerian hormone.

A delta between T3 and T0 (Final AMH Delta = fourth AMH - first AMH) was calculated, as well as a delta between T1 and T0 (Initial AMH Delta = second AMH - first AMH).

Primary variables were the medians of AMH values (ng/dL) and vaccination times. Secondary variables included age < 35 years and ≥ 35 years; EXP and NEXP; Final AMH Delta and Initial AMH Delta.

During the administration of the supplementary questionnaire and the new Informed Consent Form (ICF) via email, to recontact participants from the previous study, questions about personal history of comorbidities, medication use, surgical, gynecological, obstetric history, and habits such as alcoholism, smoking, and drug use were asked to improve the characterization of the volunteers. The researcher was available to clarify any doubts and to arrange face-to-face meetings in private conditions if requested. However, none of the participants who responded to the questionnaire sent any additional questions or requests for a face-to-face meeting. The samples from the participants who agreed to participate and were included in the present study were centrifuged for ten minutes at 3,000 rotations per minute (rpm) and the serum was stored in a -80°C freezer. Hormonal measurements were performed using the Anti-Mullerian (MRH/AMH) kit from EUROIMMUN (catalog: EQ 6161-9601).

### Statistical methods

Qualitative variables were presented by absolute and relative frequency. Variables not presenting normality by the Shapiro-Wilk test were presented by median and 95% confidence interval (CI) values. For intragroup comparison with age group and EXP and NEXP with vaccination times, the Friedman test was used. To compare between groups with age group and EXP and NEXP and vaccination times (T0, T1, T2, and T3), the Mann-Whitney test was used. The final AMH Delta and the initial AMH Delta were compared with age groups using Fisher’s Exact Test. Additionally, a Delta reduction ≤ or > 10% was considered. This value adopted was based on a previous study, that there was a significant decline in AMH levels when the second AMH decreased by more than 10% than the first AMH (Mohr-Sasson *et al*., 2022). Simple linear regression and respective confidence intervals were used to verify factors associated with the outcome variable (T3). The confidence level adopted was 95%. The statistical program used was Stata version 14.0.

### Ethical aspects

It was approved by the institutional ethics committee (CAAE: 44191821.2.0000.0082; Opinion Number: 6.077.257) and is in accordance with the Declaration of Helsinki, principles of good clinical practice, and relevant national regulations.

## RESULTS

**Figure 1:**
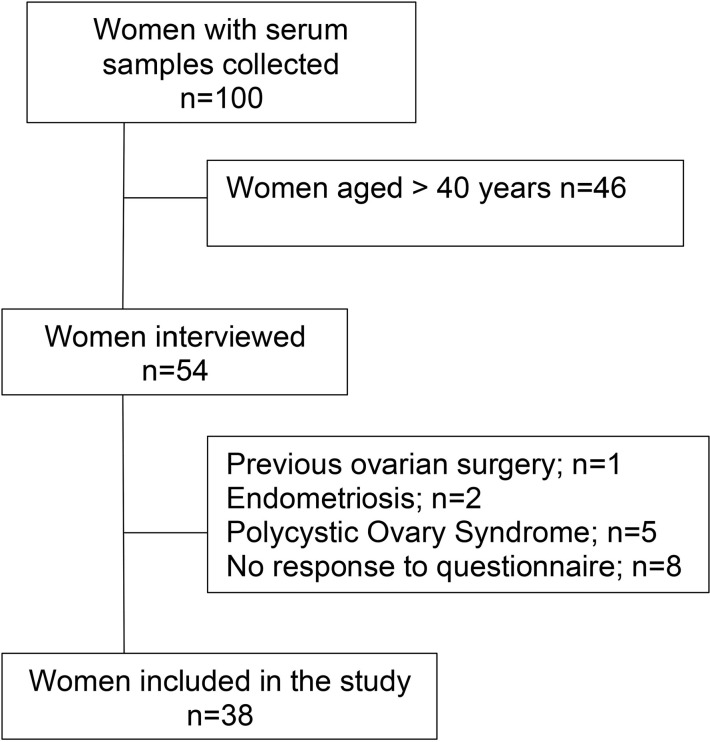
Volunteer selection flowchart.

The flowchart of the selection of volunteers from the study titled “Evaluation of humoral and T-cell-based responses after SARS-CoV-2 vaccination” for the present study is described in Figure 1.

The volunteers subjected to AMH evaluation from pre-vaccination peripheral blood collection and after the first two vaccine doses totaled 38. A total of 22 volunteers received the third vaccine dose and had AMH evaluation in the previously collected blood samples. Regarding the five patients with comorbidities, two patients reported well-controlled hypothyroidism with levothyroxine, one with gastroesophageal reflux disease, and two with fibroids. Surgical history included one patient with thyroidectomy; six with breast implants; one with cesarean section; one with rhinoplasty; one with nasal septoplasty; and one with orthopedic correction of hallux valgus. The description of the clinical characteristics of the group is in Table 2.

**Table 2:** Characterization of the group of volunteers who received vaccination against COVID-19 and underwent AMH measurement.

Variáveis	Median (p25 - 75)	Average (sd)
Age (years)	24 (22 - 30)	25.9 (5.38)
Menarche (years)	12 (11 - 13)	11.68 (1.34)
Menstrual cycle length (days)	4 (3 - 4)	3.66 (0.85)
Menstrual cycle interval (days)	28 (26-30)	28.63 (3.49)
	**n**	%
Comorbidities No Yes	33 5	86.84 13.16
Previus surgical No Yes	27 11	71.05 28.95
Contraceptive method No COC^*^ Quarterly injectable IUD† Cooper	6 18 3 11	15.79 47.37 7.89 28.95
Parity 0 1 2	35 2 1	92.11 5.26 2.63
Number of cesarean sections 0 1 2	37 0 1	97.37 0 2.63
Number of abortions 0 1	36 2	94.74 5.26
Number of children 0 1 2	37 0 1	97.37 0 2.63
Smoking No Yes	37 1	97.37 2.63
Alcoholism No Yes	38 0	100 0
Drug addiction No Yes	38 0	100 0
Exposure to SARS-CoV-2‡ No (NEXP) § Yes (EXP) ||	30 8	78.95 21.05

* COC: combined oral contraceptive.

† IUD: copper intrauterine device.

‡ SARS-CoV-2: Severe acute respiratory syndrome coronavirus 2.

§ NEXP: not exposed.

|| EXP: exposed.

**Figure 2:**
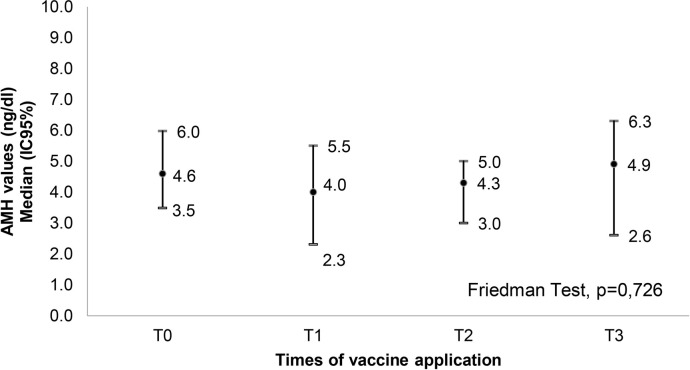
Variation in AMH values in patients who received vaccines against COVID-19.

The primary variables, median AMH values (ng/dL) in relation to the three COVID-19 vaccination times for the entire studied group, did not show a statistically significant variation, with *p*=0.726, as shown in Figure 2.

T0: pre-vaccination moment (1st AMH).

T1: 2 weeks after the first dose (2nd AMH).

T2: 2 weeks after the second dose (3rd AMH).

T3: 2 weeks after the third dose (4th AMH).

The median AMH values (ng/dL) in relation to vaccination times, considering the secondary variable age group less than and greater than or equal to 35 years, did not show a statistically significant difference within the groups less than 35 years (*p*=0.741) and greater than or equal to 35 years (*p*=0.556). However, when comparing both age groups, although the ≥ 35 years group is restricted, there was a statistically significant difference in the medians of AMH values at each vaccination time, being higher in the younger group, as described in Table 3.

**Table 3:** Comparison of average AMH values in relation to the age groups of volunteers within groups and between groups according to vaccination times for COVID-19.

Vaccine times	Age < 35 years old AMH(ng/dl)^*^ p50 (IC 95%)	n	Age ≥ 35 years old AMH(ng/dl)^*^ p50 (IC95%)	n	p^†^
T0	4.97 (3.99 - 6.54)	34	1.24 (0.14 - 2.31)	4	0.0027
T1	4.37 (2.90 - 6.39)	34	1.12 (0.39 - 1.65)	4	0.0102
T2	4.36 (3.65 - 5.36)	34	1.02 (0.15 - 1.48)	4	0.0089
T3	5.24 (2.85 - 6.48)	20	1.15 (0.48 - 1.84)	2	0.0398
p^‡^	0.741		0.556		

* AMH: Anti-Müllerian Hormone.

† Mann-Whitney Test.

‡ Friedman Test.

The evaluation of the secondary variables Initial AMH Delta and Final AMH Delta in relation to age groups considered the criterion of a Delta reduction of less than or equal to 10% and greater than 10%. In both situations, reduced ≤ 10% and reduced > 10%, there was no statistically significant difference between the age groups considering both the Initial AMH Delta and the Final AMH Delta, as shown in Table 4.

**Table 4:** Assessment of the percentage reduction in the initial Delta AMH and final Delta AMH values considering the age groups of vaccinated patients.

Age group	Initial Delta AMH (T1-T0)		*p* ^*^
	decrease ≤ 10%	decrease > 10%	
< 35 years old	18 (52.94%)	16 (47.06%)	1.000
≥ 35 years old	2 (50%)	2 (50%)	
Age group	Delta AMH final (T4 -T0)		*p* ^*^
	decrease ≤ 10%	decrease > 10%	
< 35 years old	10 (50%)	10 (50%)	0.481
≥ 35 years old	0	2 (100%)	

* Fisher's exact test.

The evaluation of the median AMH values in relation to NEXP and EXP did not show a statistically significant difference between both groups, respectively, *p*=0.738 and *p*=0.566; nor was there a statistically significant difference between the groups considering each vaccination time, as described in Table 5.

**Table 5:** Assessment of median AMH values in relation to intra and between groups of those exposed and not exposed to SARS-CoV-2 according to vaccination times.

Vaccine times	NEXP^*^	n	EXP_†_	n	p_§_
	AMH (ng/dl)_‡_ p50 (IC 95%)		AMH (ng/dl)_‡_ p50 (IC 95%)		
T0	4.83 (3.28 - 6.27)	30	4.10 (1.12 - 9.59)	8	0.6416
T1	4.03 (2.21 - 6.32)	30	3.51 (1.23 - 7.83)	8	0.6674
T2	4.31 (3.09 - 5.74)	30	3.19 (0.37 - 5.82)	8	0.2234
T3	4.65 (2.33 - 6.27)	20	6.44 (5.48 - 7.40)	2	0.2090
p||	0.738		0.566		

* NEXP: not exposed to SARS-CoV-2.

† EXP: exposed to SARS-CoV-2.

‡ AMH: anti-Müllerian hormone.

§ Mann-Whitney test.

|| Friedman test.

## DISCUSSION

The median AMH levels of the volunteers in the present study did not show a statistically significant difference at each vaccination time, from pre-vaccination to the group that received the third dose, with *p*=0.726. This reinforces both the legitimacy of vaccination, considered a necessary measure adopted in a short period, within a critical global moment in the fight against the COVID-19 pandemic, and the strengthening of the concept of no reproductive harm in women of reproductive age.

Similarly, even though not limited to AstraZeneca^®^ and CoronaVac^®^ vaccines, a systematic review of 18 studies and a meta-analysis of 14 studies, among which only two exclusively used CoronaVac^®^, highlighted that while SARS-CoV-2 infection could result in damage to ovarian reserve by reducing AMH levels, vaccination would not have this effect (Ghaemi *et al*., 2024).

A systematic review on the effect of SARS-CoV-2 infection and vaccination on ovarian reserve included 2 suggestive studies that SARS-CoV-2 infection could impair ovarian function. When comparing vaccinated and unvaccinated groups, serum AMH levels remained within the normal reserve range (>1.1 ng/dl) throughout the study period (Zhu *et al*., 2024), consistent with the presented results.

Regarding the differences in AMH values evidenced between age groups at each vaccination time, all with statistically significant differences, this is consistent with expectations. It is known that follicular depletion and consequent decrease in ovarian reserve occur with advancing age, especially from the age of 35 onwards (Vitagliano *et al*., 2023), the limit adopted for data analysis in this group of women studied.

Assessment of ovarian reserve is one way to estimate reproductive capacity. A retrospective study with vaccination restricted to CoronaVac®, investigated the effect of this measure on ovarian reserve in 46 infertile female patients with no confirmed history of SARS-CoV-2 infection. After two vaccine doses, with a one-month interval, the AMH value was compared preand post-vaccination, with a follow-up for two subsequent months, without the identification of a statistically significant difference. Additionally, it reinforced both vaccination as a rational and economical approach to protect ovarian reserve and reassured a larger number of women of reproductive age about not harming fertility (Şenkaya *et al*., 2023).

Despite the present study also considering the group not exposed to SARS-CoV-2 (NEXP), patient selection excluded the report of infertility. However, the presented data also corroborate the lack of impact on fertility and, at this moment, reinforce reproductive counseling. Contributing to the information that vaccination did not cause damage to ovarian reserve will probably not encourage further vaccination considering the end of the global pandemic announced by the World Health Organization (WHO) in 2023 (Sarker *et al*., 2023). But it potentially reassures women with reproductive desire and those undergoing infertility treatments about having already received vaccination. Reproductive counseling is part of humanization in healthcare, contributing to reducing female anxiety regarding gestational desire.

The comparison between 474 women vaccinated with CoronaVac^®^ and 474 unvaccinated demonstrated that among the unvaccinated group, there was a significant reduction in AMH values between the first and second tests. However, in subgroup analyses with age <35 and ≥35 years, this did not occur in the vaccinated group. It is worth noting that the reagent used was Elecsys^®^ AMH Plus on a Cobas 801 analyzer from Roche Diagnostics^®^ (Hunag *et al*., 2023).

Similarly, the secondary variable adopted, Initial Delta AMH, also showed a reduction in the median value of AMH after the first dose of the vaccine. Subsequently, there was a tendency for growth , both in the general group and in the subgroup analyses by age and EXP and NEXP groups, without statistically significant difference. This may be a reflection of the restricted sample of patients. Furthermore, it is assumed that part of the NEXP group had contact with COVID-19 previously asymptomatically. This would already initiate an immunological stimulus at the time of AMH measurement. Additionally, genetic responses of different populations would contribute to this transient change in AMH levels during initial vaccinations.

By adopting as the primary outcome, in women of reproductive age between 18 and 42 years, the change in AMH levels three months after the first COVID-19 vaccine, minus the initial AMH levels (AMH Delta), considering variations greater and less than 10%, a prospective study also found no significant differences (Mohr-Sasson *et al*., 2022). In the present study, reductions greater and less than 10% in AMH values, proportionally, require careful interpretation. Suggesting that the vaccine could be directly associated with this reduction greater than 10% in AMH values, paradoxically, could raise questions that vaccination led to harm to this marker. However, besides subgroup analyses requiring more critical interpretations (Wang *et al*., 2007), since the study was not initially designed for this question, the proportionality in both groups could be due to other genetic or environmental factors that also impact ovarian reserve, not exclusively limited to vaccine action.

Among these factors, a systematic review (Werner *et al*., 2024) on AMH values and modifiable lifestyle factors suggested that body mass index (BMI), smoking, oral contraceptive use, and physical activity had inversely significant associations with AMH levels. For waist-hip ratio, alcohol, and caffeine use variables, most studies found no association with this hormone. However, all effect measures of associations were heterogeneous, limiting conclusions. Patients included in the present study did not have all these lifestyle factors assessed. But the limitation of extrapolating the findings, considering a potential impact on AMH of these lifestyle-associated variables, is recognized.

Contrary to the decrease in the mean AMH value preand post-vaccination, a retrospective unicentric American cohort study, even without reporting the vaccines used and the exact time post-vaccination, included 92 women with a mean AMH value before vaccination of 4.2 ng/ml and, post-vaccination, of 5.2 ng/ml. Additionally, they did not describe which AMH assessment kits were used (Yang *et al*., 2022).

For over 20 years, attempts have been made to establish relationships between serum AMH levels and advancing age. In normovulatory patients, there is suggestive evidence of this decline over time that reinforces the role of this hormone as a marker of ovarian reserve (de Vet *et al*., 2002). If one considers patients undergoing assisted reproduction techniques (ART), such as in vitro fertilization (IVF), individualization of treatment for prediction of ovarian response could use AMH values. Thus, the best doses of gonadotropins during controlled ovarian stimulation (COS) would be indicated, providing greater safety and effectiveness (La Marca & Sunkara, 2014).

However, this is not a consensus. In a cross-sectional study using various ovarian reserve biomarkers as possible predictors of response to COS, it was suggested that none of them, including AMH, showed good predictive capacity. An exception was the Ovarian Response Prediction Index (ORPI) for hyper-response in normo-ovulatory infertile women (Peluso *et al*., 2021). Similarly, AMH also did not demonstrate predictive capacity for clinical pregnancy rate in patients undergoing assisted reproductive technology (ART). However, it should be noted that the same sample was used for AMH level analysis with five different kits: Immunotech®, Beckman Coulter II Gen. RUO®, Beckman Coulter II Gen. IVD®, Ansh®, and Elecsys Roche®. The conclusion emphasized that pregnancy rates varied greatly with the same samples from the same AMH patients, depending on the kit used. Thus, different AMH values could lead to misguided clinical decisions in ART (Liss *et al.,* 2017).

In this context, 12,917 women were monitored with four types of AMH tests (Immunotech I®, Beckman Coulter II RUO®, Generation II with IVD certification - BCII®, and Ansh Labs I®), identifying that the distribution of AMH concentration was heterogeneous after age adjustment among the assays, and that the rate of AMH decline with age varies for each test used (Plociennik *et al.,* 2018).

The differences observed may reflect that the AMH kits used represent different proportions of the four serum isoforms of AMH, primarily represented by a precursor hormone, proAMH, its amino and carboxy-terminal portions, respectively AMHC and AMHN, and a non-covalent binding AMHN,C. Inconclusive evidence suggests that conversion predominantly occurs in the ovary dependent on pro-protein convertase subtilisin/kexin-type 3 (PCSK3) and PCSK5, hormone-dependent action, and still not fully understood physiology. This makes the process and understanding of clinical use of AMH more complex than imagined. While the recognition of different isoforms for AMH is intriguing and adds complexity to its pathophysiology, it also encourages more caution in its clinical use, requiring further research (La Marca, 2019).

The need for an international standard for AMH and better validation of available assays, as well as the search for conversions to adapt different kits in the interpretation of AMH values, are barriers to be overcome. However, studies using only one AMH kit can contribute to this process to maximize the clinical utility of this very promising biomarker for ovarian function and, consequently, ovarian reserve (Dewailly *et al.,* 2014).

The present study used only one kit for the evaluation and follow-up of all patients (EUROIMMUN). This reduces the bias resulting from finding AMH values influenced by different proportions of AMH isoforms by different kits. Additionally, it contributes to advancing understanding of AMH value and the impact of vaccination as it is a single-center cohort study, in which hormonal collection and measurement methods were similar for each volunteer.

In this study, women in the menacme who received the vaccine were included, with no clear correlation associated with infertility, a frequent topic of research using AMH. Considering the ethical principles of the Helsinki Declaration, even patients with low AMH levels identified in this research were informed that available studies do not determine a serum AMH threshold value associated with live birth rates and, primarily, the lack of data on its predictive value in women attempting to conceive without ART (Peigné *et al.,* 2023). Thus, rigorously, the external validity of the results of this study is limited to recognizing that, in a short period that included two to three vaccine doses, using the same AMH kit for follow-up, there was no reduction in AMH in the studied group. However, considering the need for rapid vaccine development and deployment in enormous quantities, a signal of no reproductive harm is promising and may encourage further research for larger follow-ups of these patients.

However, the lack of identification of damage to ovarian reserve suggests that even pregnant women over 35 years of age, as well as patients with known decreased AMH levels, would have the protective benefit of vaccination. This action have reduced the prevalence and impact of infectious diseases, underpinning global health security and social well-being. Thus, maintaining high vaccination coverage is crucial. Threatening these goals by vaccine hesitancy or refusal of vaccines, in despite of the availability, endangering the success of vaccination programs, is unacceptable (Vigezzi *et al*., 2024).

Limiting factors include a small convenience sample, which may affect the generalizability of the results, the relatively short follow-up time, the lack of sample power calculation, and the fact that vaccination occurred with more than one type of COVID-19 vaccine (AstraZeneca® and CoronaVac®). However, this reflects a practical situation that occurred in much of the Brazilian population, namely, receiving the available vaccine at the time. Thus, the decision not to perform more subgroups for analysis considering the use of one or two vaccines is justified, as it would limit interpretation due to the type I error trend.

The rigor in patient selection in a cohort study, the exclusive use of one AMH kit, and the importance of reassuring reproductive counseling, considering initial research after the sanitary and humanitarian crisis, with recently declared termination due to a global pandemic, are factors that enhance the present findings.

## CONCLUSION

Vaccination against COVID-19 with the AstraZeneca® and CoronaVac® vaccines did not indicate any detriment to anti-Müllerian hormone levels in premenopausal women.
